# Towards Flexible and Low-Power Wireless Smart Sensors: Reconfigurable Analog-to-Feature Conversion for Healthcare Applications

**DOI:** 10.3390/s24030999

**Published:** 2024-02-03

**Authors:** Mikhail Manokhin, Paul Chollet, Patricia Desgreys

**Affiliations:** C2S Team, ComElec Department, Laboratoire de Traitement et Communication de l’Information (LTCI), Télécom Paris, Institut Polytechnique de Paris, 19 Place Marguerite Perey, 91120 Palaiseau, France; mikhail.manokhin@telecom-paris.fr (M.M.); pachollet@telecom-paris.fr (P.C.)

**Keywords:** analog-to-feature converter, low power, wireless smart sensors, non-uniform wavelet sampling, feature selection, arrhythmia detection, human activity recognition, Gm-C integrator

## Abstract

Analog-to-feature (A2F) conversion based on non-uniform wavelet sampling (NUWS) has demonstrated the ability to reduce energy consumption in wireless sensors while employed for electrocardiogram (ECG) anomaly detection. The technique involves extracting only relevant features for a given task directly from analog signals and conducting classification in the digital domain. Building on this approach, we extended the application of the proposed generic A2F converter to address a human activity recognition (HAR) task. The performed simulations include the training and evaluation of neural network (NN) classifiers built for each application. The corresponding results enabled the definition of valuable features and the hardware specifications for the ongoing complete circuit design. One of the principal elements constituting the developed converter, the integrator brought from the state-of-the-art design, was modified and simulated at the circuit level to meet our requirements. The revised value of its power consumption served to estimate the energy spent by the communication chain with the A2F converter. It consumes at least 20 and 5 times less than the chain employing the Nyquist approach in arrhythmia detection and HAR tasks, respectively. This fact highlights the potential of A2F conversion with NUWS in achieving flexible and energy-efficient sensor systems for diverse applications.

## 1. Introduction

The modern world witnesses an increasing scientific interest in wireless sensor networks (WSNs): networks of multiple spatially dispersed independent sensors that monitor some environmental and physical data and collectively transmit them to a central station (aggregator) [1]. The fast development of such networks and the Internet of Things (IoT) leads to the need for new context-aware smart sensors deployed in numerous fields, such as precision agriculture [2], industry [3], transportation [4], security [5], education services [6], and healthcare [7]. However, these emerging applications impose certain design constraints: such sensors should remain reliable, compact, and cheap, with a lifetime of several years. In particular, reducing energy consumption in wireless smart sensors in order to improve their autonomy is one of the most challenging and necessary tasks.

In some applications, where anomalies or events are detected, the processing is performed on the captured signal to extract characteristic information from it, i.e., “features”, which can then be used by classification or regression algorithms. The conventional wireless sensor approach implies that both feature extraction and classification are performed at the aggregator level after receiving the samples sent from sensors. In this case, the signals are acquired at sampling frequencies based on the Shannon–Nyquist theorem, allowing the complete signal information to be maintained. Due to a relatively high Nyquist rate, the transmission of all samples constitutes a significant part of the sensor’s total consumption budget. When only certain specific information contained in the signal is helpful for the detection task, this approach turns out to be energetically inefficient. Hence, minimizing the volume of data transmitted from the sensor to the aggregator is critical.

For this purpose, several architectures of analog-to-information (A2I) conversion have been proposed to implement a well-known compressive sampling (CS) technique [8], which relies on the fact that most real signals can be considered sparse or compressible after transformation into some domain. CS combines acquisition and compression processes and allows the original signal to be recovered from this compressed data with fewer measurements than in traditional methods. Nonetheless, it exhibits a constrained compression ratio [9] and performs a complete signal reconstruction, which remains superfluous in the previously mentioned applications focused on detecting anomalies or events. Moreover, complex sparse recovery algorithms are required to rebuild a sparse signal from an undersampled set of measurements [10,11], which entails severe energy and time costs.

On the contrary, analog-to-feature (A2F) conversion is capable of further reducing the quantity of transmitted data. To accomplish this task, it only extracts useful features relevant to the specific task from the analog signal [12] directly in the sensor node. These features are then digitized and transmitted to a remote aggregator or serve as inputs for machine learning (ML) algorithms for classification within the sensor. The first method performs the anomaly or event detection at the aggregator. At the same time, the second allows for more efficient frugal communication by sending only the classification results and, thus, reduces the consumption due to wireless transmission. Nevertheless, the implementation of the classifier at the sensor level will increase its power consumption. The A2F approach facilitates the deployment of a low-power IoT as the entire communication chain is relieved regarding the quantity of sent data and required throughput. For a successful implementation of A2F conversion, one should define, for a given application, the relevant features for extraction and how to extract them.

One of the drawbacks of current A2F converter solutions is that they are designed for specific applications [13,14]. In this paper, extending the findings presented in [15], our objective is to design a generic, reconfigurable A2F converter suitable for processing several types of signals with low sampling frequencies (below hundreds of kilohertz). Our work follows the one carried out in [16], where the A2F conversion approach has been applied for binary arrhythmia detection in electrocardiogram (ECG) signals, outperforming alternative acquisition techniques (conventional Nyquist rate sampling and CS) in terms of power efficiency and hardware simplicity. Effectively, ECG wearable sensors allowing for cardiac activity measurement represent an example of sensors constituting body area sensor networks (BASNs), which are strongly constrained in the energy available for sensor consumption.

Usually, the arrhythmia detection process consists of four main consecutive steps: data collection, noise removal, feature engineering, and classification [17]. Feature engineering involves the extraction of specific features from the ECG waveform. Some studies use raw signals due to the increasing popularity of deep learning (DL) methods [18], while others identify fiducial points such as QRS-complex, ST-segment, R-peak, and P- and T-waves. Many traditional signal processing approaches have been proposed for feature engineering, such as wavelet transform and its variations, methods based on time domain and mathematical morphology, and derivative-based techniques, to name a few. Among ML approaches, we can enumerate mode decomposition, K-nearest neighbors (KNNs), naïve Bayes (NB), and support vector machine (SVM). Once the required features have been extracted, the last stage classifies the ECG signal with the help of traditional or ML approaches. The first group includes, for example, threshold-based techniques, principle component analysis, multi-model decision learning, etc. Whereas SVM, decision tree (DT), random forest (RF), convolutional neural network (CNN), long short-term memory (LSTM), and plenty of other methods belong to ML approaches.

Herein, we adopted the A2F converter’s architecture based on the non-uniform wavelet sampling (NUWS) [19] to perform the feature extraction and selected arrhythmia detection as the first application for the developed converter.

The evolution of IoT has enabled the deployment of numerous edge devices equipped with inertial sensors. These sensors provide researchers with collections of unprocessed physiological signals suitable for analyzing human activities [20]. In addition to ECG sensors, these inertial sensors, including accelerometers and gyroscopes, can also be a part of power-constrained BASN. Human activity recognition (HAR) using such signals has emerged as a highly explored domain [21], driven by the growing demand for diverse human-centric applications such as smart home systems, sports tracking, and health monitoring [22,23].

Like arrhythmia detection, motion sensor-based HAR systems perform four principal steps, but one may require additional pre-processing between denoising and feature extraction, e.g., to separate body and gravitational acceleration [24]. The three primary feature extraction and selection methods are classical hand-crafted features, automatic feature generation through DL methods, and their hybrid. The produced features are fed to a traditional ML or DL model for classification. Traditional ML methods, including DT [25], SVM [26], RF [27], KNN [28], and the hidden Markov model [29], have already achieved remarkable success in HAR by surpassing the classification accuracy of DL techniques for specific datasets. However, CNN models, which obviate the need for hand-crafted feature extraction [30], have taken the lead in this field [31]. A novel approach proposed in [32] transforms human activities into a high-level feature space with highly interpretable categorical features whose combinations are unique across activities. The classification problem is thereby broken into multiple sub-problems and a combination task. The principal advantage of this method is its extensibility to new activities and datasets.

Given that HAR is a typical pattern recognition task, we introduce it as a second application of the designed A2F converter to prove its universality and suitability for diverse sensing use cases. In order to reduce the amount of transmitted data, the developed converter will extract only a handful of relevant features from the input signal. As a result, using simple classifiers should suffice, eliminating the need for CNN models. The relevant features for extraction that are defined during the classifiers’ simulations for both applications will serve as a basis for the complete circuit design.

In summary, the main contributions of this paper are as follows: (1) the expansion of simulation results for both applications and derived hardware specifications based on relevant extracted features; (2) the choice of appropriate circuit solutions for elements constituting the A2F converter; (3) the design and simulation of a G_m_-C integrator at the schematic level; (4) the estimation of energy consumption showing the advantages of the developed approach.

The rest of the paper proceeds as follows. In Section 2, we introduce the architecture of the developed A2F converter and the details of feature extraction and selection processes. Section 3 provides the results of training and evaluating the classifiers for our applications, which are then used in Section 4 to deduce the specifications for the converter’s hardware implementation. Section 5 presents the simulations of a G_m_-C integrator executed at the schematic level. The power consumption of this circuit is used next in Section 6 to estimate the energy consumed by the communication chain employing the A2F converter. Section 7 summarizes our research conclusions and perspectives for further work.

## 2. Analog-to-Feature Conversion

### 2.1. Reconfigurable Architecture

**Figure 1:**
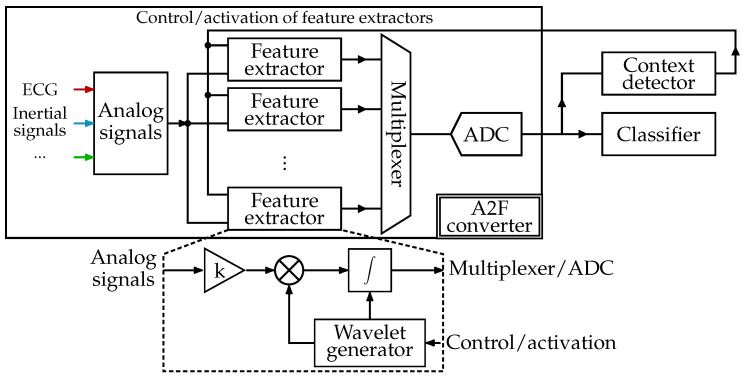
Architecture of the acquisition system with the reconfigurable A2F converter based on the NUWS feature extraction.

The architecture of the envisioned intelligent acquisition system is presented in Figure 1. It contains the generic, adaptable A2F converter inspired by the feature-extracting ADC (analog-to-digital converter) architecture described in [33]. Unlike the interpretable statistical “features”, generally derived from the signal in time (pulse amplitude, mean value, standard deviation, slope, timing information, etc.) or frequency (principal frequency, spectral energy, magnitude of components from fast Fourier transform, etc.) domain [34,35], we will extract the features meaningless for humans but relevant for the classification procedure. In summary, our acquisition system performs the operations listed below:

- Extraction of analog domain features based on the NUWS technique [19] with the help of several parallel feature extractors.
- Conversion of extracted features from analog to digital domain.
- Application-specific binary or multiclass classification.
- Context detection, which activates the necessary feature extractors and adjusts their internal wavelet generators according to the nature of the input processed signal, as well as the type and desired accuracy of classification.

Moreover, classification algorithms will probably offer a notable benefit in A2F converters, mitigating design constraints on analog circuits. This advantage will be possible if classification algorithms can learn and accommodate eventual nonlinearities introduced by analog circuits, such as amplifiers and filters, without compromising the classification process. In contrast, traditional processing methods assume linearity across all components.

### 2.2. Feature Extraction

The NUWS technique, first proposed as a CS method for acquiring radio frequency signals [19], was chosen for feature extraction. Wavelet functions are commonly used in signal processing, data compression, and other applications. By definition, a wavelet ξ is a square-integrable, zero-mean, oscillating, centered around zero function with finite, bounded support with nonzero values. NUWS involves non-uniformly sampling the wavelet transform coefficients of the observed signal, which includes conducting a continuous wavelet transform of the input signal x(t) followed by non-uniform sampling. The continuous wavelet transform Wx is performed by multiplying the input signal by a wavelet and integrating the result over the wavelet support duration, as described below:

(1) $$Wx\left(a,b\right)=\int^{R}x\left(t\right)\;\cdot \;\xi_{a,b}^{*}\left(t\right)\;dt\;\mathrm{with}\;\xi_{a,b}\left(t\right)=\frac{1}{\sqrt{a}}\;\cdot \;\xi \left(\frac{t-b}{a}\right),$$

where $\xi_{a,b}^{*}\left(t\right)$ denotes the conjugate of $\xi_{a,b}\left(t\right)$, ξ is a mother wavelet (Haar, Gabor, Morlet...), a>0 is a scale factor providing the size of the wavelet support, and b∈R defines the temporal position.

From the perspective of electronic circuitry, NUWS-based feature extraction corresponds to mixing the analog signal with tunable wavelets and subsequently integrating them within the analysis window, as illustrated at the bottom of Figure 1. One of the primary advantages of the NUWS is that its continuous wavelet transform enables obtaining temporal and frequency information of the input signal at once. However, according to the Heisenberg uncertainty theorem, the simultaneous resolution in time and frequency is limited [36]. As the wavelet support widens, one improves the precision in the frequency domain at the cost of less precise information in time and, conversely, for shorter support.

**Figure 2:**
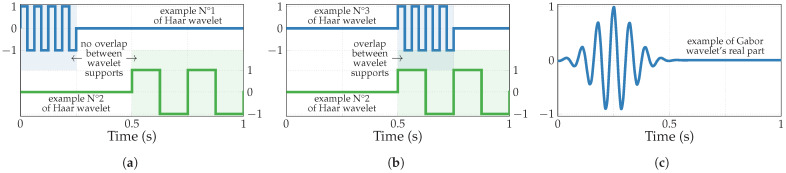
Examples of wavelets: (**a**) two non-overlapping and (**b**) two overlapping Haar wavelets; (**c**) the real part of the Gabor wavelet.

In this study, two principal wavelet families that can be realized in an analog manner, namely Haar and Gabor wavelets, are utilized to implement NUWS-based feature extraction. Examples of these wavelets are shown in Figure 2. Haar wavelets [37] represent a family of square functions that alternate between 1 and −1 within their support, turn to 0 elsewhere, and are straightforward to generate. On the other hand, Gabor wavelets, rather challenging for a generation with tunable parameters, result from the multiplication of a complex exponential by a Gaussian window.

In contrast to definition (1), which implies that the oscillation frequency and the support size of the wavelet are correlated (inversely proportional), we make them independent. Thus, our transform becomes more flexible, offering three degrees of freedom when generating wavelets $\psi_{f_{o},t_{s},\Delta t}$, including the oscillation frequency $f_{o}$, support size $t_{s}$, and temporal position (time shift) Δt within a fixed-size analysis window of the signal. Mathematically, the extracted feature *F* is then expressed as:

(2) $$F\left(f_{o},t_{s},\Delta t\right)=\int_{\Delta t}^{\Delta t+t_{s}}x\left(t\right)\;\cdot \;\psi_{f_{o},t_{s},\Delta t}\left(t\right)\;dt$$

Given that each of these three quantities can take an infinite number of values, the size of the resulting wavelet dictionary containing all possible wavelets of the same family is potentially infinite. Accordingly, the number of features that can be extracted is also limitless. In this regard, for every application explored with the A2F converter, initial dictionaries of both Haar and Gabor wavelets are constructed in a manner that reduces their size and potential redundancy. For example, the process of building Haar wavelet dictionaries occurred as follows. From the lowest frequency corresponding to one wavelet period (1, −1 oscillation) in the analysis window, the oscillation frequency $f_{o}$ has been doubled up to the frequency equal to half of the dataset’s sampling frequency (since half of Haar wavelet’s period cannot be shorter than one interval between consecutive samples of the input signal). For each $f_{o}$, we considered different support sizes $t_{s}$, starting from the one containing only one entire wavelet period and multiplying this size by two until reaching the full analysis window length. To avoid overlapping between wavelets with the same $f_{o}$ and $t_{s}$, we allowed only the time shifts Δt that are multiples of $t_{s}$.

Nonetheless, the quantity of Haar and Gabor wavelets, i.e., ultimately features, generated using this method remains exceedingly large. For instance, in our binary arrhythmia detection application, the initial dictionaries contain 502 Haar and 2534 Gabor wavelets. Hence, a proper feature selection procedure becomes indispensable to identify a reduced set of features that are relevant for a particular classification task.

### 2.3. Feature Selection

Aiming to choose only a few useful features from all produced by wavelet dictionaries, we perform a sequential forward search (SFS) algorithm, which belongs to a group of wrapping methods of feature selection and serves as a dimensionality reduction technique. SFS provides adapted solutions with improved classification performance for a specific classifier and a given subset of available features. It constructs the classifiers with different subsets of these features as inputs and assesses their performance. As demonstrated in Algorithm 1, which represents the basic SFS, the target set *S* is initially empty. For every new subset $S\cup \{F_{i}\}$, a newly trained classifier is evaluated. The target set *S* is gradually filled with the features $F_{i^{\prime }}$ that yield the highest classification metric value (accuracy ACC).
**Algorithm 1** Basic sequential forward search.1:S←∅                                                                    ▹ Target feature set *S* is initially empty2:**while** $\left|S\right|\leq N_{F,max}$ **do**                     ▹$N_{F,max}$ is a maximum number of selected features3:    **for all** $F_{i}\notin S$ **do**                                                 ▹ Test each feature that is not yet in *S*4:        $ACC_{i}\leftarrow ACC\left(S\cup \left\{F_{i}\right\}\right)$5:    $i^{\prime }\leftarrow \mathrm{arg max}\left(ACC_{i}\right)$6:    $S^{\prime }\leftarrow S\cup \left\{F_{i^{\prime }}\right\}$7:    **if** $ACC\left(S^{\prime }\right)>ACC\left(S\right)$ **then**    ▹ Condition check and else block are optional and                                                              only required to obtain ever-increasing accuracy8:        $S\leftarrow S^{\prime }$9:        $ACC\left(S\right)\leftarrow ACC\left(S^{\prime }\right)$10:   **else**11:        **break**

However, the SFS algorithm’s complexity scales quadratically with the number of available features. Hence, to reduce significantly its execution time, we perform the pre-selection of the top 100 features produced by the initial wavelet dictionary. For this purpose, without constructing an ML model, this number of features is chosen according to the information gain (IG) criterion [38]. For a given dataset *D*, this supervised filter method of feature selection ranks each feature *F* by its IG value expressed as:

(3) $$IG\left(D,F\right)=H\left(D\right)-\sum_{i=1}^{N_{F}}\frac{\left|D_{i}\right|}{\left|D\right|}H\left(D_{i}\right)\;\mathrm{with}\;H\left(D_{\left(i\right)}\right)=\sum_{j=1}^{N_{C}}-p_{(i)j}\;\cdot \;\log_{2}\left(p_{(i)j}\right),$$

where $N_{F}$ is the number of possible values of feature *F*, $D_{i}$ corresponds to a subset from *D* with feature *F* taking the value $F_{i}$, $\left|D_{i}\right|/\left|D\right|$ denotes the portion of examples from *D* with $F=F_{i}$. $H(D_{\left(i\right)})$ stands for a Shannon entropy of a whole dataset *D* or a subset $D_{i}$, where $p_{(i)j}$ is the portion of examples in $D_{\left(i\right)}$ belonging to a class *j*, and $N_{C}$ is the total number of classes. To make our case suitable for this expression operating with discrete values of features, we divided each feature’s range of continuous values into $N_{F}=12$ equal intervals.

Given the inherent focus of the basic SFS algorithm on solely maximizing the classification accuracy, it is amenable to modification to accommodate the hardware complexity and energy consumption of the A2F converter. Thus, the adapted SFS limits the available number of parallel extractors since each extracts only those multiple features that do not overlap in the same analysis window (e.g., produced by two non-overlapping Haar wavelets from Figure 2a). Furthermore, the optimized SFS additionally takes into consideration the energetic cost associated with the extraction of each feature.

Since we extract NUWS-based features from the analog signal segments, only the feature space reduction techniques designed for single-vector-based ML problems have been studied. The techniques dedicated to the sequential data, such as the one presented in [39] and based on HMM, feature stacking, and LDA, are not applicable here.

**Figure 3:**
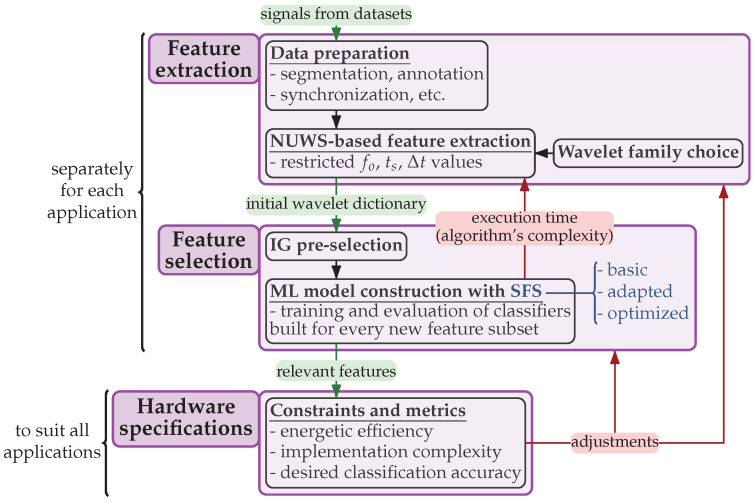
Overall methodology applied throughout the study.

To summarize, a visualization of the overall methodology applied throughout this study is shown in Figure 3. It involves the following three main steps: performing the feature extraction and feature selection separately for each studied application and defining hardware specifications that suit all applications to build a generic A2F converter.

## 3. Healthcare Applications

In this section, we first describe the datasets used to simulate both ECG arrhythmia detection and HAR. Then, through training and evaluating classifier models, we define the pertinent extracted features and the system specifications, enabling the complete circuit design. We apply the methodology of constructing a classification model outlined in Section 2. However, to enhance the efficiency of ML models, particularly neural networks (NNs), and speed up calculations using graphics processing units (GPUs), we re-implemented the ML algorithms previously realized in Matlab^®^ R2021b [16] using Python 3.10 with TensorFlow 2.9.2 library.

### 3.1. Description of Datasets and Classification Metrics

Table 1 summarizes the details of datasets and corresponding issued data used for simulations of both applications. Anomaly detection in ECG signals, more precisely binary arrhythmia detection, has been performed on the single channel (modified limb lead II, obtained by placing the electrodes on the chest) signals from the MIT-BIH arrhythmia dataset [40] that is widely used by researchers to evaluate their classifiers. Its original ECG recordings were acquired by Del Mar Avionics model 445 reel-to-reel Holter recorders and then played back on a Del Mar Avionics model 660 playback unit for digitization. This dataset contains 48 ECG recordings of 30 min each, taken from 47 patients and sampled uniformly at 360 Hz with 11-bit resolution over a 10 mV range. To maintain a standard 70/30% proportion between training and test sets, as is typical in ML problems, 34 recordings were used to generate features for a training set and 14 for a test set. To detect arrhythmia in each heartbeat, the length of the analysis window has been set to 256 samples. This corresponds to 711 ms and, thus, approximately to the duration of one annotated heartbeat.

We chose the UCI-HAR dataset [41] for HAR simulations, which is commonly used in this field and is already divided into analysis windows for training and test sets. It comprises 3-axial linear acceleration and angular velocity signals, sampled at a constant rate of 50 Hz. A smartphone (Samsung Galaxy S II, manufactured by Samsung Electronics Co., Ltd., headquartered in Suwon, Republic of Korea) with an embedded accelerometer (Kionix KXTF9, manufactured by Kionix, Inc., based in Ithaca, NY, USA) and gyroscope (TDK InvenSense MPU3050, manufactured by TDK InvenSense Corporation, located in San Jose, CA, USA) was worn on the waists of 30 volunteers performing three static and three dynamic activities of daily living (ADLs)—sitting, standing, laying, walking, walking upstairs, and walking downstairs. The obtained data were randomly partitioned into training and test sets, also with a 70/30% proportion. After pre-processing with noise filters, the recordings were divided into sliding analysis windows, 128 samples long (2.56 s), with a 50% overlap.

For the binary arrhythmia detection, as two main classification metrics, we use accuracy, which is simply a proportion of correctly predicted instances, and sensitivity (recall), i.e., the ability to recognize the heartbeats with arrhythmia among truly abnormal instances:

(4) $$Accuracy=\frac{TP+TN}{TP+TN+FP+FN},\;Sensitivity=\frac{TP}{TP+FN},$$

where TP, TN, FP, and FN stand for true positive (with arrhythmia), true negative (without arrhythmia), false positive, and false negative, respectively. The specificity (selectivity) that represents the proportion of correctly classified observations without arrhythmia among truly normal observations is used as well:

(5) $$Specificity=\frac{TN}{TN+FP}$$

Since there are more than two classes in the UCI-HAR dataset, we consider two methods of ADLs classification: multiclass and one-vs-all binary HAR. The latter requires six distinct classifiers to differentiate each specific activity from the others. For the multiclass HAR, we use accuracy as the classification metric. Meanwhile, a strong imbalance of occurrences of each class in the UCI-HAR dataset while performing one-vs-all binary HAR forces us to use the Matthews correlation coefficient MCC. Accounting for the size of all four categories of the confusion matrix (TP, TN, FP, and FN), it is more informative than the accuracy in evaluating binary classification problems with imbalanced datasets [42]:

(6) $$MCC=\frac{TP\;\cdot \;TN-FP\;\cdot \;FN}{\sqrt{\left(TP+FP)(TP+FN)(TN+FP)(TN+FN\right)}}$$

### 3.2. Arrhythmia Detection in ECG Signals

Binary arrhythmia detection simulations in this work have exclusively employed Haar wavelets despite a slight loss in classification accuracy compared to Gabor wavelets, as confirmed in the complete study performed in [16]. Since Haar wavelets are straightforward to generate digitally, they simplify the structure of the wavelet generator and mixer when designing the A2F converter.

**Figure 4:**
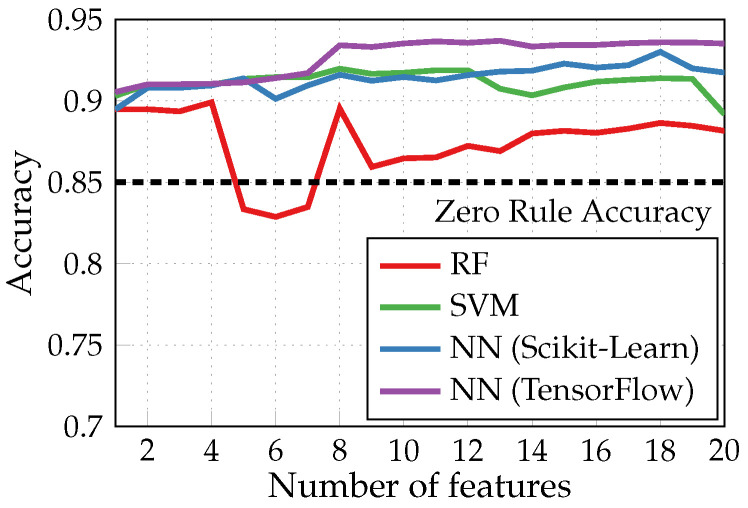
Accuracy of binary arrhythmia detection performed by different classifiers without the SFS feature selection.

Figure 4 demonstrates the results obtained with three different types of classification models built without using SFS feature selection after the IG algorithm: random forest (RF), support vector machine (SVM), and artificial neural network (NN). The latter was built with one hidden layer of 10 neurons using scikit-learn and TensorFlow for comparison. Both are software ML libraries available in Python. However, among other differences, scikit-learn is a higher-level one with a broader range of models. In contrast, TensorFlow is more of a low-level library implied for use with NNs and allows to take advantage of GPUs for more efficient training. Here (and in the following figures), we plot the accuracy (or other metrics) against the number of chosen features. The accuracy of a zero rule classifier, which attributes to every tested example the most frequent class present in the test set (85% of heartbeats in the test set are without arrhythmia), is shown as a reference. The NN classifier built and trained with TensorFlow provided the best performances; hence, this configuration was chosen for further simulations with the SFS algorithm.

**Figure 5:**
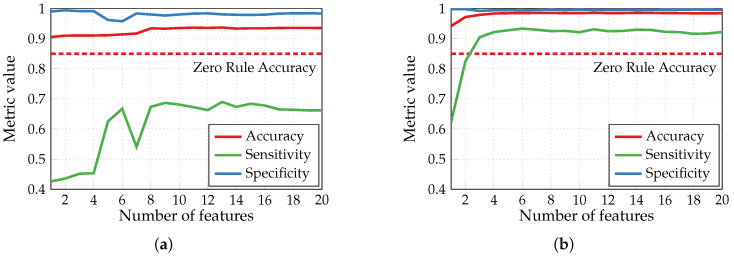
Impact of basic SFS algorithm on binary arrhythmia detection performed by NN classifiers (built with TensorFlow): (**a**) feature selection only with IG; (**b**) feature selection with IG + basic SFS.

Figure 5 shows the impact of the basic SFS algorithm on the accuracy, sensitivity, and specificity of binary arrhythmia detection performed by NN classifiers with one hidden layer of 10 neurons trained during 1500 epochs. Sensitivity is a crucial metric for such a classifier, and it achieves around 93% using the basic SFS, while the feature selection only with IG provides less than 69% sensitivity.

**Figure 6:**
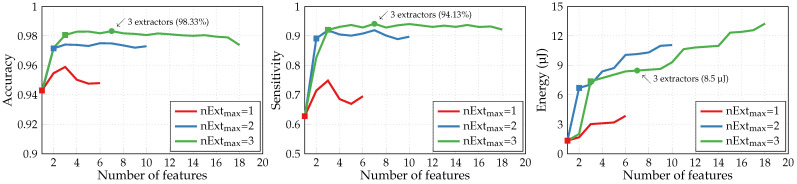
Metrics of binary arrhythmia detection performed by NN classifiers with adapted SFS.

Figure 6 illustrates the performances produced by the adapted SFS algorithm with three different values of a maximum number of parallel extractors $nExt_{max}$. For each curve, the square marker shows the point when the maximum number of extractors $nExt_{max}$ is reached. Limited to three feature extractors, the algorithm achieves a 98.33% accuracy and a 94.13% sensitivity with seven extracted features and 8.5 μJ energy consumption (green round marker in Figure 6). Similar results have been previously obtained in [16], where the extraction of six features by three extractors also entailed the consumption of 10.9 μJ to achieve a 98.4% accuracy. Thus, this new validated simulator can be employed further for our second application of the A2F converter.

### 3.3. Human Activity Recognition

Static ADLs (sitting, standing, and laying) are naturally characterized by relatively constant acceleration and angular velocity values. Such signals integrate to zero with any wavelet, which is an inherently zero average function. Subsequently, these features are unable to distinguish static activities from each other. Hence, we introduced an additional “constant” wavelet with a unitary value across the entire analysis window to the initial wavelet dictionaries. Furthermore, we do not need the wavelet generator to extract features produced by this wavelet since the concerned signal is directly integrated within the whole analysis window and results in a value proportional to its average.

**Figure 7:**
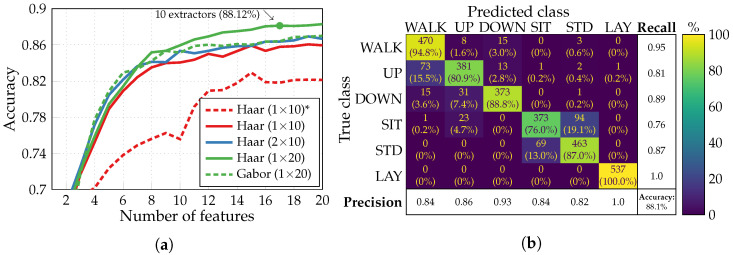
Multiclass HAR performance with basic SFS algorithm: (**a**) accuracy achieved by NN classifiers of different structures (*—classifier with default initializers); (**b**) confusion matrix of the preferable dictionary-classifier configuration.

Figure 7a illustrates the accuracy of multiclass HAR obtained with the basic SFS algorithm by NN classifiers of different structures: one hidden layer of 10 neurons, two hidden layers of 10 neurons, and one hidden layer of 20 neurons. To improve the classification accuracy further, we switched the kernel and bias initializers of the hidden layer from the default “glorot uniform” and “zeros”, respectively, to “random uniform”. The only example utilizing the default initializers in Figure 7a is plotted as a red dashed curve. It corresponds to an NN classifier with one hidden layer of 10 neurons trained with Haar wavelets. Gabor wavelets, though less straightforward to generate, are generally more effective than Haar wavelets. However, they yield a significantly lower classification accuracy in our specific case, as shown by the green dashed curve. Consequently, we will not proceed with their usage further. The preferable configuration (Haar wavelets, NN classifier with one hidden layer of 20 neurons) achieves an 88.12% accuracy with 17 features extracted by 10 extractors (green round marker). Its confusion matrix with color mapping based on recall (sensitivity) is presented in Figure 7b. Since it is a multiclass classification, the sensitivity represents the proportion of instances of a given class that are correctly predicted. The precision, i.e., the fraction of relevant instances among those predicted as a given class, is provided below the confusion matrix for complementary information. Quite a substantial confusion between sitting and standing ADLs can be noticed due to similar local shapes of inertial measurements’ time series corresponding to these static activities.

**Figure 8:**
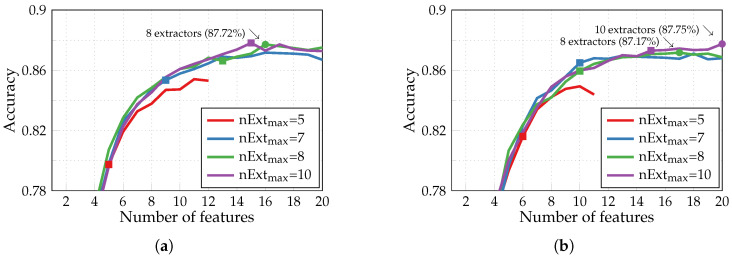
Accuracy of the multiclass HAR performed by NN classifiers with the adapted SFS algorithm and features generated from (**a**) acceleration and angular velocity signals; (**b**) acceleration signals.

Executing the multiclass HAR with adapted SFS for the identical preferable dictionary-classifier configuration produces the accuracy curves depicted in Figure 8a. On all four curves with different values of a maximum number of extractors, the square marker again indicates the point when this maximum $nExt_{max}$ is reached. Notably, the case with $nExt_{max}$ set to 10 yields a lower accuracy of 87.8% (with 15 extracted features) compared to the basic SFS with 10 extractors as well (green round marker in Figure 7a). The reason is that when training an NN classifier, the parameters are initialized randomly. It impacts how different feature subsets perform and which features are added afterward to the target set. Furthermore, by decreasing the number of extractors to eight, we can extract 16 features to achieve a slightly lower classification accuracy of 87.72% (green round marker in Figure 8a). In this particular case, out of the six available inertial signals, only x- and y-axes of acceleration and z-axis of angular velocity produce the required features. And since solely two features are extracted from the gyroscope, a similar simulation has been conducted but with acceleration signals alone producing the features. According to the results presented in Figure 8b for $nExt_{max}=8$, we can restrict our use to a single type of inertial sensor—an accelerometer—at a moderate loss in classification accuracy (down to 87.17%).

Although binary classification is less common in the HAR field than its multiclass counterpart, we also performed all the required simulations for one-vs.-all binary HAR. However, it requires six different NN classifiers and more features and extractors in total to distinguish all six activities from each other, as shown in Table 2, where the best trade-off points of all binary and multiclass classifiers are summarized. All classifications except DOWN-vs-ALL necessitate features generated with the help of the “constant” wavelet. Hence, apart from regular extractors with wavelet generators, they require those implementing the direct integration of the input signal within the analysis window.

While our multiclass HAR classifier provides a lower accuracy on the UCI-HAR dataset compared to state-of-the-art CNN models (see Table 3), it requires at least 102 times fewer parameters and a 23 times smaller input size. We can implement this simple feedforward NN with still digital architecture directly within the sensor rather than at the aggregator level. It will help further cut down on energy usage due to the wireless transmission by sending exclusively the classification results. Furthermore, employing analog memristor-based, reconfigurable NN [43,44,45,46] will enable digitizing strictly the classifier’s decision while providing the application- or context-specific adaptation. The current order of HAR classification accuracy may suffice for non-critical applications. However, if necessary, it is still possible to enhance accuracy and reliability by acquiring additional types of signals and enabling data fusion [47] and distributed event detection [48]. Feature stacking has yet to be considered and may also be applied in future works to improve classification performance by combining the predictions from several ML models.

### 3.4. Discussion

According to the results obtained with the adapted SFS in Figure 6, to achieve a 98.33% accuracy in the case of binary arrhythmia detection, our A2F converter, instead of the 256 samples required for a uniform Nyquist rate sampling, uses only seven features extracted from the entire period of the ECG signal (modified limb lead II) with the help of three parallel extractors. It corresponds to a 97.3% compression ratio. As for the multiclass HAR, the extraction of 16 or 17 relevant features from three inertial signals composed of 384 = 3 × 128 samples with the help of eight parallel extractors (Figure 8) results in a 95.8% or 95.6% compression ratio and 87.72% or 87.17% classification accuracy.

Nevertheless, several assumptions and simplifications have been made during simulations, and potential limitations of the proposed approach have to be taken into account. First of all, the signals issued from both datasets were already preprocessed with noise filters. One of the future research directions is to test the developed classifier with raw signals. Also, the integration performed during simulations represents an approximation of real integration produced in hardware.

For arrhythmia detection, as indicated in Section 3.1, only the signals from one lead (modified limb lead II) of possible 12 leads have been used for the training and evaluation of our classifiers, potentially limiting the applicability of the proposed approach. Moreover, we have assumed a strict synchronization of each annotated heartbeat segment within the analysis window (see Table 1). Although not yet implemented in this study, the required synchronization system for analog QRS-complex detection and proper R-peak placement can be borrowed from the existing ultra-low-power designs [56,57,58].

In the context of HAR, it is essential to acknowledge the limitations imposed by the analyzed UCI-HAR dataset. These constraints include a restricted variety of ADLs and low representativeness due to ranges of values, such as stride frequency and stair step height, limited by the experiment conditions. Additionally, we must address multiple heterogeneities arising from various possible software, hardware, and data collection configurations in inertial motion-based HAR systems [24]. At the same time, the signals in the UCI-HAR dataset have been collected from a particular body location by a specific smartphone with a specific operating system and embedded sensors. Furthermore, although HAR application scenarios highlight the potential of the A2F converter’s implementation in real-time systems [59], i.e., performing fall detection or sports assistance, usage of the UCI-HAR dataset so far has limited our study to offline models due to a long minimum response time imposed by the analysis window size equal to 2.56 s. Hence, subsequent investigations may focus on adequately reducing the analysis window length based on the duration of corresponding human activities [60] and analyzing another suitable dataset, such as the recently released multimodal wearable sensors-based CSL-SHARE dataset [61], to study the developed system’s real-time perspective.

## 4. Hardware Implementation

The ongoing work deals with the complete circuit design of the developed A2F converter based on the relevant features for extraction defined during the simulations for both applications. In this section, we deduce the hardware specifications and review the state-of-the-art solutions for each block to choose the appropriate technology node and architectures that meet the defined specifications.

### 4.1. Required Specifications

**Figure 9:**
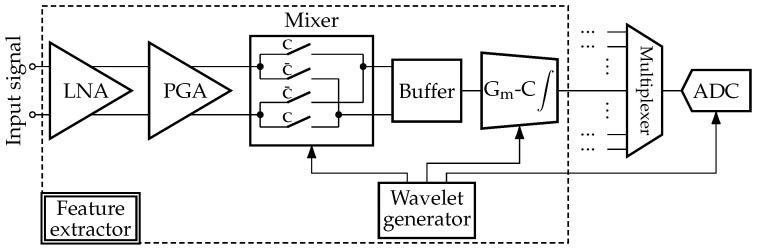
Hardware implementation of the feature extraction chain.

As a part of the intelligent acquisition system shown in Figure 1, our generic A2F converter requires eight parallel feature extractors to accommodate both explored applications. While only three are sufficient for binary arrhythmia detection, for multiclass HAR, we need all eight, albeit two of them do not require the presence of a wavelet generator in their composition to execute the direct integration. For the sake of simplicity, Figure 1 does not illustrate the multiplexers at the input of feature extractors needed to choose the relevant inertial signals while performing the HAR task. A possible hardware implementation of the feature extraction chain, including its principal components as the differential amplification stage, the analog mixer, the G_m_-C integrator, and the ADC, is displayed in Figure 9. One lever to reduce the required size of the converter’s implementation and the energy consumed during the feature extraction process is to bring the amplification stage outside the extractors and dedicate a separate amplification stage for each signal. Hence, the amplifier will not be activated at once in several extractors during the extraction of features produced by the overlapping wavelets from the same signal.

To process at least two types of studied signals—single-channel ECG recordings and three outputs of inertial sensors—the amplification stage should have a variable gain. Nevertheless, if commercially available 3-axis analog output accelerometers (as well as a z-axis gyroscope if needed), for example, from analog devices, are used to provide inertial measurements for the HAR task, amplification may not be necessary, as their outputs are ratiometric to reference voltages ranging from 1.8 V to 6 V. Designing the converter with Haar wavelets, first and foremost, facilitates the implementation of wavelet generators, enabling their entirely digital structure. Additionally, it simplifies the analog mixer to four switches based on complementary metal–oxide–semiconductor (CMOS) transmission gates when the differential amplification stage is used. The integrator, functioning basically as a first-order low-pass filter (LPF), should possess a cut-off frequency considerably lower than the minimum frequency of the slowest signal to be integrated. From the analysis of the extracted features in Figure 6 and Figure 8 (green round markers), these frequencies are 5.63 Hz and 1.56 Hz for arrhythmia detection and multiclass HAR, respectively. However, to ensure the eventual possibility of extracting any other feature, we need $F_{c}\ll 1/2.56\;s=0.39$ Hz corresponding to the slowest Haar wavelet with one period in the HAR analysis window.

Up to this point, all the simulations presented in Section 3 utilized 64-bit double-precision floating-point data. Nevertheless, the analog features we extract are converted into a digital domain to reduce the quantity of data for subsequent classification, whether directly within the sensor or after transmission to an aggregator. For arrhythmia detection application, it was found that 6-bit precision is sufficient to keep the classification accuracy at the same level as obtained with the unquantified data [16].

**Figure 10:**
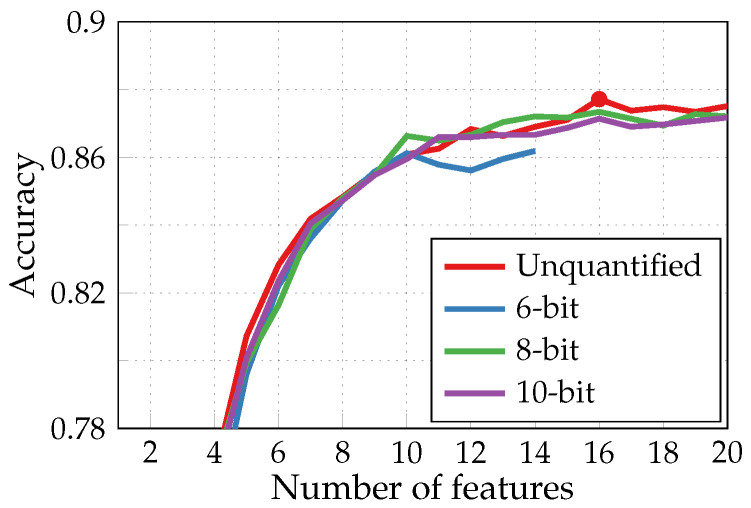
Impact of the quantification level on the accuracy of the multiclass HAR performed by NN classifiers with adapted SFS algorithm ($nExt_{max}=8$).

Below, we demonstrate the results of a similar study for HAR to determine the necessary specifications for the ADC. Figure 10 presents the impact of the quantification level on the accuracy of the multiclass HAR classification performed by NN classifiers with the adapted SFS algorithm limited to eight parallel extractors and with features generated from acceleration and angular velocity signals (see green curve in Figure 8a). The training and test sets provided for NN classifiers were composed of features quantified after the pre-selection by the IG criterion. The choice of features by the SFS algorithm and, thus, the classification accuracy are inevitably affected by the level of quantification since some features no longer supply relevant information for classification. In contrast to the arrhythmia detection task, for a 6-bit precision, a noticeably lower accuracy is achieved with respect to unquantified cases, and even more extractors are required to accommodate more than 15 features. Conversely, an 8-bit precision already achieves an accuracy of a similar order as without quantization, despite the variability of classification performances. Such fluctuations are induced by the process of random initialization of weights and biases while training a new NN classifier each time we increase the number of input features.

The required ADC conversion rate depends on the maximum oscillation frequency of extracted features, which will not exceed half of the datasets’ sampling frequencies (25 Hz or 180 Hz). The reason arises from the fact that one period of the fastest Haar wavelet is twice the interval between consecutive samples of the input signal. Accordingly, the A2F converter under development has relaxed ADC speed requirements. Table 4 summarizes the hardware specifications of its principal components.

### 4.2. State-of-the-Art Solutions

As an initial consideration, the amplification stage of a biosensing front-end application-specific integrated circuit (ASIC) implemented in a 180 nm CMOS process and proposed in [62], optimized for low noise, power, and area, meets our requirements. Its reconfigurable amplifier comprises a low-noise amplifier (LNA) and a programmable gain amplifier (PGA) and provides a variable gain from 38 dB to 72 dB. Table 5 presents a comparison of its performance with several other state-of-the-art designs. The amplification stage elaborated on in [62] stands out from the rest with its considerably small area and a wider range of gain variation, but its power consumption significantly exceeds that of the most recent designs, especially of [63,64].

The 10-bit charge redistribution successive-approximation register (SAR) ADC with a 40 kHz maximum sampling frequency and a 0.3 μW power consumption proposed in [62] satisfies our needs as well. However, in scenarios where ADC constitutes a significant portion of the overall power consumption in the A2F converter, it is possible to find a less power-hungry design with reduced but sufficient speed, given the low required conversion rate for our applications, or even to modify the existing state-of-the-art SAR ADC circuit for this purpose.

Implementing a first-order LPF with a sub-Hertz cut-off frequency poses a significant challenge. Nevertheless, Table 6 presents a few examples of designs found in the literature that seem to fulfill our specifications. The solution proposed in [68] is basically an improved version of a widely tunable G_m_-C integrator designed in [69]. Despite providing relatively low power consumption, dimensions, and a wide range of cut-off frequency flexibility, the filter of [68] still presents two possible issues. First, its lowest achievable cut-off frequency is too close to the frequency of the slowest Haar wavelet (0.39 Hz), which, as explained in Section 4.1, may eventually be required to extract the features from inertial signals during the HAR task. Second, a buffer is necessary to pass from the mixer’s differential output to the filter’s single-ended input, as shown in Figure 9. Despite a higher minimum cut-off frequency and increased power consumption as opposed to [70], the circuit in [68] occupies a significantly lower area, provides a larger input dynamic range, and uses a 180 nm CMOS technology as well as the chosen amplification stage from [62] and the digital wavelet generator synthesized in [16]. Hence, in Section 5, we perform the simulations at the schematic level of the G_m_-C integrator based on the design elaborated in [68] with an aim to achieve a very low cut-off frequency but without unnecessary tuning.

Regarding the choice of technology node for the entire converter’s circuit implementation, we opt for a 180 nm CMOS process. Based on the data presented in Table 5 and Table 6, this process is still widely employed in many modern analog designs due to its maturity, availability of design tools, and optimal balance between performance and cost-effectiveness.

## 5. Gm-C Integrator Design

**Figure 11:**
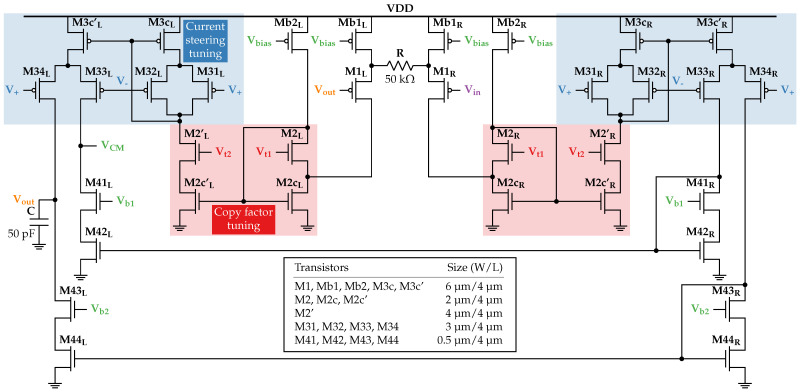
G_m_-C integrator schematic based on the design in [68].

In this section, we present the simulations of the first-order LPF (G_m_-C integrator) based on the design in [68] and define its biasing and control voltages to achieve a cut-off frequency low enough for the explored A2F converter’s applications. The schematic of the G_m_-C integrator is shown in Figure 11 with all transistors’ dimensions. It is based on a classic mirrored operational transconductance amplifier (OTA) with a degenerated PMOS input differential pair. The minimization of the cut-off frequency in this circuit is achieved either by maximizing the loading capacitance or by minimizing the OTA’s transconductance. While the technology limitations might restrict the former (we set it to 50 pF), the latter is adjusted in this circuit with the help of two techniques. The first one, copy factor tuning, allows controlling the amounts of complementary currents copied from the input stage through the NMOS high swing cascode current mirror (transistors M2, M2c, and M2c’) by setting $V_{t2}\leq V_{t1}$. The second technique, current steering tuning, splits the current passed through the 1:1 PMOS high swing cascode current mirror (transistors M31, M32, M3c, and M3c’) into two complementary currents flowing through transistors M33 and M34. The differential voltage $V_{gc}$ adjusts the complementary gate voltages $V_{\pm }=V_{c}\pm V_{gc}$ and, therefore, the fractional values of these output currents.

With a very low sub-Hz cut-off frequency being the primary purpose of using this G_m_-C integrator in our case, we would like to simply choose the $V_{t2}$ and $V_{gc}$ values that minimize the OTA’s transconductance. Basically, it means that both adjustment techniques should reduce the output current flowing in the integrator’s output branch made of transistors M34, M43, and M44. Thus, we should decrease $V_{t2}$ and increase $V_{gc}$ as much as possible. However, the OTA becomes more asymmetrical as $V_{t2}$ and $V_{\pm }$ deviate from $V_{t1}$ and $V_{c}$, respectively, degrading the integrator’s dynamic range and noise performance. Therefore, the optimal pair of $V_{t2}$ and $V_{gc}$ values should be chosen. Since the integrator in [68] was designed in a 180 nm CMOS technology from TSMC^®^, which differs from targeted Xfab^©^ technology, and several biasing voltages are also not stated in the paper, we present below the simulations at the schematic level to define all the necessary and missing voltage values and assess the integrator’s performance.

**Figure 12:**
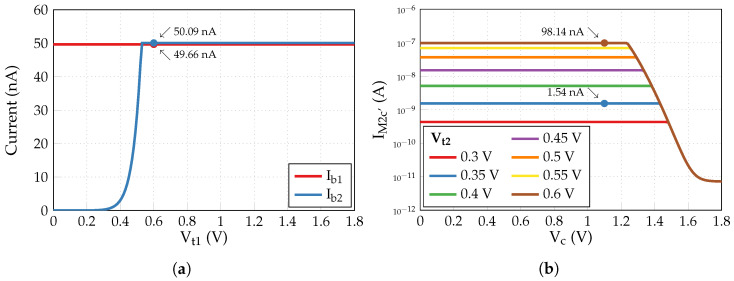
Results of DC simulations in the open-loop configuration to define (**a**) $V_{t1}$ and (**b**) $V_{c}$.

First, DC simulations of the circuit in the open-loop configuration are performed to define proper $V_{t1}$ and $V_{c}$ values. According to Figure 12a, $V_{t1}=0.6\;V$ is enough to keep the currents $I_{b1}$ and $I_{b2}$ approximately equal to a 50 nA biasing current introduced through a 1:1 current mirror to Mb1 and Mb2 (as well as to maintain M2 and M2c in the subthreshold region). Next, with the current steering tuning off ($V_{gc}=0V$⇒$V_{\pm }=V_{c}$), a constant common voltage $V_{c}$ is swept from 0 V to 1.8 V with $V_{t2}$ varied in a 0.35–0.6 V range (see Figure 12b). For further simulations, $V_{c}$ is set to 1.1 V value, ensuring a correct constant current sunk by M2c’ for any $V_{t2}\leq V_{t1}$.

**Figure 13:**
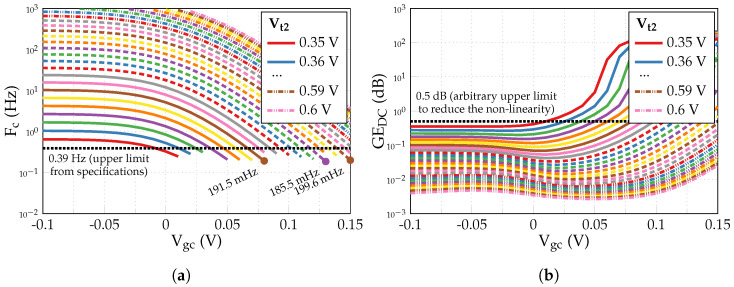
Results of AC simulations in the integrator configuration with a common-mode voltage $V_{CM}=0.9\;V$ to define $V_{t2}$ and $V_{gc}$ voltages: (**a**) cut-off frequency $F_{c}$; (**b**) DC gain error $GE_{DC}$.

Figure 13 presents the results of AC simulations in the integrator configuration with a common-mode voltage $V_{CM}$ initially set to 0.9 V, which is half of the power supply voltage VDD, as in [68]. $V_{t2}$ and $V_{gc}$ are swept from 0.35 V to 0.6 V and from −0.1 V to 0.15 V, respectively, while biasing voltages $V_{b1,2}=V_{t2}+0.1\;V$ ensure the correct functioning of the output branches. Figure 13a illustrates the obtained cut-off frequencies $F_{c}$, where only the points corresponding to $V_{t2}$, $V_{gc}$ pairs from Figure 13b with a DC gain error $GE_{DC}$ below 0.5 dB (the arbitrarily chosen value to reduce the non-linearity) are shown. According to the specifications mentioned in Section 4.1, we require $F_{c}\ll 0.39\mathrm{Hz}$. Thus, the three best $V_{t2}$, $V_{gc}$ pairs of the $V_{CM}=0.9\;V$ case with the lowest cut-off frequencies are summarized in Table 7.

**Figure 14:**
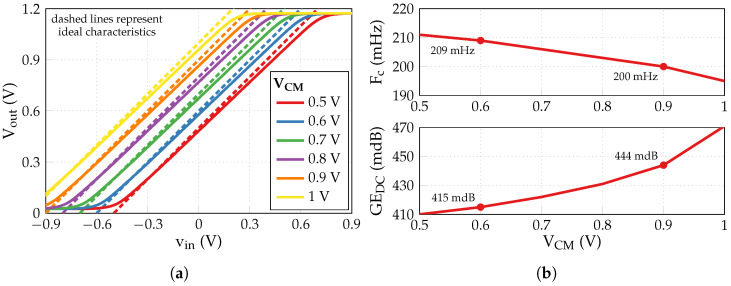
Impact of common-mode voltage $V_{CM}$: (**a**) $v_{in}$–$V_{out}$ characteristic; (**b**) cut-off frequency $F_{c}$ and DC gain error $GE_{DC}$.

The pair with $V_{t2}=0.5\;V$ and $V_{gc}=0.15\;V$ displays a good trade-off between the cut-off frequency and the DC gain error, providing $F_{c}=199.6\;\mathrm{mHz}$ and $GE_{DC}=0.444$ dB. It is used to produce static input–output characteristics shown in Figure 14a, where the output voltage $V_{out}$ is plotted against the “differential” input voltage $v_{in}=V_{in}-V_{CM}$ for a range of common-mode voltage $V_{CM}$ values. As seen, the maximum $V_{out}$ is limited to ≲ 1.17 V, making it reasonable to switch to $V_{CM}=0.6\;V$ for the sake of higher input symmetry and dynamic range. Although decreasing $V_{CM}$ leads to a slight increase of $F_{c}$ up to 209 mHz, it also results in the reduction of $GE_{DC}$ down to 0.415 dB (see Figure 14b). The results of AC simulations have been reproduced with $V_{CM}=0.6\;V$, providing three similar $V_{t2}$, $V_{gc}$ pairs with comparatively close performances to those obtained previously with $V_{CM}=0.9\;V$ in terms of $F_{c}$ and $GE_{DC}$, as reflected in Table 7.

**Figure 15:**
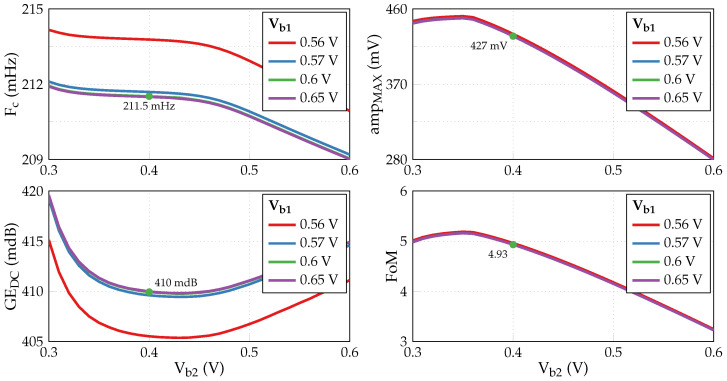
Impact of biasing voltages $V_{b1}$ and $V_{b2}$.

Keeping the same control voltages $V_{t2}=0.5\;V$ and $V_{gc}=0.15\;V$, we carried out AC and DC simulations with $V_{b1}$ and $V_{b2}$ sweep to determine their optimal values. Figure 15 illustrates the results, where two new characteristics are introduced. A maximum input amplitude $amp_{MAX}$ stands for the minimum value between minimum (negative) and maximum (positive) “differential” input voltages that ensure $\partial V_{out}/\partial V_{in}$ deviation from ideal unity gain below 0.5 dB. We also define a figure of merit that should be maximized:

(7) $$FoM=\frac{amp_{MAX}}{F_{c}\;\cdot \;GE_{DC}},$$

since we aim to minimize $F_{c}$ and $GE_{DC}$ but simultaneously increase the input dynamic range. It is seen that $V_{b1}$ values ≳0.6 V have a negligible impact on either characteristic. Meanwhile, setting $V_{b2}=0.4\;V$ puts us close to an optimum in terms of FoM. Table 8 summarizes the chosen biasing and control voltages required for the functioning of the circuit and its performance characteristics. Since our choice of control voltages reduces the transconductance and thus the currents in the output stages of the G_m_-C integrator, its estimated power consumption *P*, calculated as a product of the sum of currents and the supply voltage, decreases from 1.08 μW to 625 nW. In the next section, this revised value will serve to estimate the energy spent by the communication chain with the A2F converter.

The hardware implementation of other elements composing the A2F converter is under development. However, unlike the integrator and wavelet generator, these circuits are not critical for the consumption estimation of the communication chain. Hence, we can use their power consumption from the existing state-of-the-art designs for this purpose.

## 6. Consumption Estimation

In this section, we compare the energetic efficiency of our communication chain to those based on Nyquist and A2I techniques to show the benefits of using the A2F converter in wireless sensors, at least for two explored applications. We estimate the energy consumption of the acquisition systems based on these approaches, followed by the identical low-energy classic transmission system in each case.

**Figure 16:**
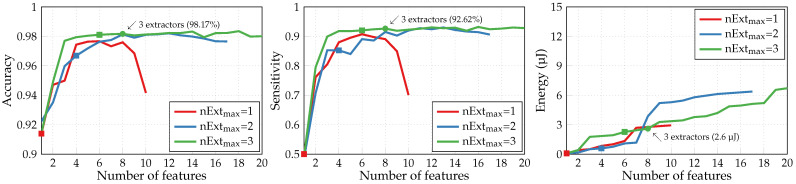
Metrics of binary arrhythmia detection performed by NN classifiers with optimized SFS.

So far, to define the relevant extracted features, we trained and evaluated the NN classifiers with the adapted SFS algorithm, which maximizes the classification accuracy for a given limited number of available parallel extractors $nExt_{max}$. However, as indicated in Section 2.3, the optimized SFS can additionally take into account the energetic cost of each extracted feature. In binary arrhythmia detection, this algorithm manages to considerably reduce the required energy consumption while maintaining relatively high values of classification metrics, as seen in Figure 16. Limited to three feature extractors, it achieves a 98.17% accuracy and a 92.61% sensitivity with eight extracted features and 2.6 μJ energy consumption (green round marker). Whereas, with the adapted SFS, seven features and a significantly higher energy of 8.5 μJ are required for moderately improved accuracy and sensitivity of 98.33% and 94.13%, respectively (green round marker in Figure 6). Applying the optimized SFS algorithm to the HAR, however, will necessitate the modification of its original evaluation criterion; otherwise, the resulting choice of extracted features leads to a severe limitation of achievable classification accuracy in favor of energy reduction.

Hence, further estimations of energy consumption induced by using the A2F converter in the acquisition chain will be based on the results of optimized SFS with eight extracted features shown in Figure 16 (green round marker) for arrhythmia detection and of adapted SFS with 16 and 17 features illustrated in Figure 8a,b (green round markers, yet not showing the energy required for the extraction of chosen features) for HAR. The energetic cost of each extracted feature is calculated as follows:

(8) $$E_{A2F}^{feat}=\left(P_{amp}+P_{int}+P_{wavelet}\right)\Delta t+\frac{P_{ADC}}{F_{s}},$$

where $P_{amp}$, $P_{int}$, $P_{wavelet}$, and $P_{ADC}$ stand for the power of the amplification stage, the integrator, the wavelet generator, and the ADC, respectively; Δt is wavelet support, i.e., the duration of a wavelet producing the corresponding feature; $F_{s}$ represents a sampling frequency of the ADC. The power equal to 5.04 μW of the state-of-the-art amplification stage from [62] considered in Section 4.2 and composed of LNA and PGA is used as $P_{amp}$. Up to this point, the G_m_-C integrator’s power equal to 1.08 μW has been used to calculate the energetic cost of extracted features (Figure 6 and Figure 16). However, we proceed further with an updated value $P_{int}=625\;nW$ obtained from the simulations in Section 5. Next, the estimated power consumption of the digital wavelet generator with clock gating synthesized in Xfab^©^ CMOS 180 nm technology [16] serves as $P_{wavelet}$. SAR ADC, also demonstrated in [62], provides $P_{ADC}$ and $F_{s}$ equal to 0.3 μW and 40 kHz, respectively.

The acquisition chain representing the conventional Nyquist approach considered here is entirely composed of the elements presented in [62] and operates at $F_{s}=2\;\mathrm{kHz}$. Alongside the previously mentioned amplification stage and SAR ADC, it involves an anti-aliasing G_m_-C type third-order LPF preceding the ADC and consuming 0.7 μW. Thus, its total power consumption is 6.04 μW.

Similar to [16], for the binary arrhythmia detection application, we also consider the A2I architecture proposed in [9], designed for the CS acquisition of biological signals. In the case of ECG signals, this A2I converter has been configured to work with 32 channels and 128 sampling cycles in the analysis window, corresponding to a compression ratio of 4 compared to the Nyquist approach. SAR ADCs within each channel provide a 10-bit precision as well. For a sampling frequency $F_{s}$ of 2 kHz, the total power consumption of the circuit is 0.9 μW. Nonetheless, this does not account for the power required to recover the original signal from the compressed data.

The wireless transmission system considered for all three approaches uses a Bluetooth low energy (BLE) standard and consumes 3.7 nJ per transmitted bit [71], i.e., 37 nJ per sample with a 10-bit precision.

**Figure 17:**
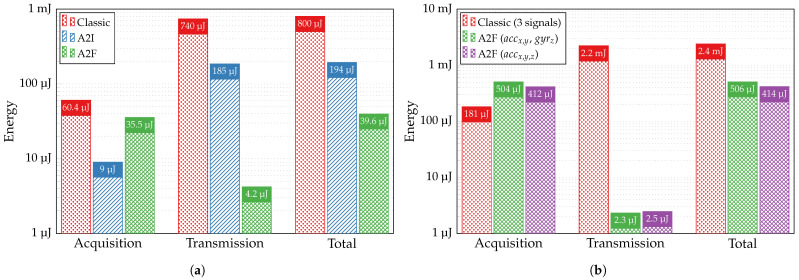
Energy required to process 10 s of (**a**) ECG and (**b**) inertial signals with different wireless sensor approaches.

We can first re-estimate the energy required to extract the relevant features from 10 s of the ECG signal and transmit them from the sensor to the aggregator for further classification, taking into account the updated performance of the binary arrhythmia detection classifier and the reduced power consumption of the G_m_-C integrator. Figure 17a then compares it with the energy necessary for acquiring and transmitting the ECG signal of the same duration but using alternative wireless sensor techniques, i.e., classic (Nyquist) and A2I (CS). The proposed communication chain with the A2F converter, in total, consumes 20.2 and 4.9 times less than those employing classic and A2I approaches, respectively. Whereas the consumption attributed to the transmission process in the A2F converter is 44 times lower than that of the A2I converter, the energy spent in the acquisition chain represents 89.4% of total consumption in the A2F approach, and its value exceeds the acquisition part in the A2I converter by 3.9 times.

Performing similar calculations for the HAR, we obtain the breakdown of energy consumption, as shown in Figure 17b, induced by processing 10 s of the inertial signals with the Nyquist and A2F approaches. Both cases of the A2F conversion represented as green round markers in Figure 8—with features extracted from signals of two types ($acc_{x}$, $acc_{y}$, $gyr_{z}$) and only from accelerometer signals ($acc_{x}$, $acc_{y}$, $acc_{z}$)—are compared to the classic approach that samples three signals with $F_{s}=2\;\mathrm{kHz}$ and sends them to the aggregator. As observed, employing the A2F converter leads to a substantial decrease in the overall energy consumption of wireless sensors by 4.7 or 5.8 times, owing to the drastic reduction in energy associated with transmission. Since the acquisition part in the A2F approach of both applications remains high and even exceeds that of the classic approach while used for the HAR task, the development of a modified version of the optimized SFS algorithm—a power-aware feature selection based on the complete simulations of the A2F converter’s circuit—is necessary.

So far, we considered sending the extracted features from the sensors for further classification at the aggregator level. However, as indicated in Section 3.3, we can perform the classification directly within the sensor to diminish the amount of transmitted data further and send only the classification results. For this purpose, it is possible to implement at least a digital architecture of our simple feedforward NNs realized on a field programmable gate array (FPGA) [72,73,74], in an ASIC [74,75], or on a microcontroller [76,77].

**Figure 18:**
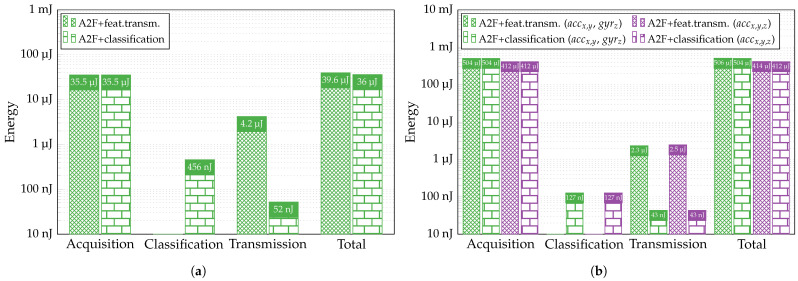
Energy required to process 10 s of (**a**) ECG and (**b**) inertial signals with different A2F approaches.

In [72], a multilayer perceptron with one hidden layer and a 7-6-5 topology was implemented in Xilinx Artix-7^®^ FPGA. The proposed design consumes $P_{FPGA}=120\;mW$ and requires $t_{class}=270\;ns$ for classification. Without re-evaluating the data, due to a similarity of the topology with our NNs, we can use these values to estimate the energy spent on processing 10 s of analyzed signals with the classification performed directly within the sensor. While the acquisition part remains unchanged, we spend additional energy due to classification and reduce the consumption attributed to the transmission process. The former is calculated as a product of the required power $P_{FPGA}$, the time necessary for one classification $t_{class}$, and the number of classifications performed in 10 s. In binary arrhythmia detection, instead of sending eight features coded on 10 bits, we transmit only one bit of the classification result, thus reducing by 80 times the energy of transmission. In the multiclass HAR, for both cases ($acc_{x}$, $acc_{y}$, $gyr_{z}$; $acc_{x}$, $acc_{y}$, $acc_{z}$), we transmit three bits (to accommodate six classes) per classification rather than 16 or 17 features coded on 10 bits, which results in a 53 or 57 times reduction, respectively. However, as seen in Figure 18, despite a considerable decrease in transmission energy, the total consumption reduction is negligible, especially in the HAR case. For both applications, the acquisition process now represents more than 98% of the sensor’s total consumption. It means that optimization should be conducted within the acquisition and feature extraction circuitry to cut down substantially on the overall energy usage in sensors.

We can also compare the performance of our system employing the in-sensor classification (without considering the transmission energy) to recent embedded designs for both studied applications. An ECG processor ASIC with arrhythmia detection, proposed in [78] and implemented in a 65 nm CMOS technology, consumes 2.04 μW. It locates R-peaks and abnormal R-R intervals by searching for local extremes of the signal derivative with self-adaptive thresholds, achieving a 96.9% classification sensitivity on the MIT-BIH Arrhythmia dataset. The authors improved the classification sensitivity to 98.2% in the ASIC proposed in [79] and designed for long-term implantable cardiac monitoring. Within the measured total power, which increased to 2.04 μW due to more functions onboard, the detection part consumes only 81.9 nW. A wearable ECG processor for arrhythmia detection presented in [80] and fabricated in a 110 nm CMOS technology provides an average classification accuracy of 97.34% with a 4.08 μW total power consumption. It utilizes a Hilbert transform-based R-peak detection, a Haar discrete wavelet transform to extract features, and a hybrid classifier that combines a linear pre-classifier and a polynomial kernel SVM. At the same time, our A2F converter shows a 98.17% accuracy and a 92.62% sensitivity with an estimated average power consumption of 3.55 μW while detecting arrhythmia.

A wearable HAR system designed on the Spartan-6 FPGA in [81] uses a PCA-based dimensionality reduction technique to choose the relevant features extracted from time and frequency domains and a two-hidden-layer backpropagation NN for classification. However, it was tested using accelerometer data from another dataset, namely PAMAP, achieving an 89.2% accuracy and consuming 268 mW. A HAR framework implemented on the TI-CC2650 microcontroller unit in [82] shows 97.7% accuracy in identifying six activities and their transitions in the online training experiment with nine users while consuming 11.24 mW during computation. The fast Fourier transform and discrete wavelet transform features have been extracted from stretch and accelerometer sensors for further inference and training using an NN. The authors followed up in [83] with the first fully integrated custom hardware accelerator implemented in a 65 nm TSMC^®^ technology that achieves a 95% accuracy in recognizing eight human activities while consuming 51 μW of active and 14 μW of idle power. A deep NN used the extracted features similar to the previous work for classification. In comparison, the proposed A2F converter distinguishes six human activities with an estimated average power consumption equal to 50.4 μW (features from $acc_{x}$, $acc_{y}$, $gyr_{z}$) or 41.2 μW ($acc_{x}$, $acc_{y}$, $acc_{z}$) and an 87.7% or 87.2% accuracy.

Thus, for both applications, certain embedded designs offer better energetic efficiency, especially considering the rough consumption estimates made for the A2F converter components. Nevertheless, the advantage of the developed solution lies in its genericity, meaning that it will suit several application cases and remain relatively low-power. Further reduction of energy spent in our acquisition chain can be achieved by employing analog, reconfigurable NNs [44,45,46] based on memristors to digitize the classification result solely. This would also enable learning algorithms such as backpropagation to be implemented on a chip [43], providing the application or context-specific adaptation in the A2F converter and probable mitigation of design constraints by learning and accommodating nonlinearities introduced by analog circuits.

## 7. Conclusions and Perspectives

In this study, we demonstrate the benefits of utilizing an A2F converter for event detection in wireless smart sensors in terms of reduced throughput, power consumption, and hardware complexity. For this purpose, a generic and reconfigurable architecture has been proposed, capable of extracting only relevant features from various types of low-frequency signals directly in the analog domain. The extraction of features based on the principle of NUWS is followed by digitization and event classification.

After successfully reproducing the simulation results of classification for already-proposed binary arrhythmia detection using more advanced software, we explored a second application, namely, HAR. Based on the results obtained, we have defined the hardware specifications required for the physical implementation of the converter. After reviewing the available state-of-the-art circuit designs for each principal element constituting the A2F converter and choosing among them the appropriate solutions meeting our specifications, we performed the design and simulation of a G_m_-C integrator at the schematic level to achieve a required low cut-off frequency without unnecessary tuning present in the original design.

For both explored applications, we estimated the energy consumed during the acquisition and transmission of the corresponding signals in the wireless sensor’s communication chain, showing the advantages of the developed A2F conversion over alternative acquisition techniques. In upcoming work, we plan to expand the review of state-of-the-art solutions to include other blocks that compose the proposed A2F converter and refine its total consumption. All elements of its architecture are to be designed at the schematic and layout levels using the Cadence^®^ tools. Upon completing all necessary simulations and verifications, we will attempt to develop a modified version of the optimized SFS algorithm. Its power-aware feature selection based on the converter’s circuit simulations is intended to account for its total measured consumption and analog circuit non-idealities. Thereafter, the chip fabrication process will begin, followed by the physical demonstration of the A2F converter’s performance.

A 180 nm CMOS process was chosen as the technology node for the overall converter’s circuit implementation. However, as intelligent sensors become more prevalent, more digital components tend to be incorporated at the sensor level. Therefore, transitioning to a smaller, more advanced technology node that enables the design of faster, more compact, and power-efficient digital circuits may be reasonable. 

## Figures and Tables

**Table 1:** Setup for the A2F converter simulations.

Application	Arrhythmia Detection	Human Activity Recognition
Dataset (signals)	MIT-BIH Arrhythmia [40] (single channel from 48 ECG recordings of 30 min each, sampled at 360 Hz)	UCI-HAR [41] (3-axial acceleration and angular velocity signals from a waist-mounted smartphone, sampled at 50 Hz)
Classes	2 (normal, abnormal)	6 (walking, upstairs, downstairs, sitting, standing and laying)
Initial dict. size:		
– Haar wavelets	502	248 × 6 = 1488
– Gabor wavelets	2534	552 × 6 = 3312
Type of learning	supervised learning, 70/30% proportion between training and test sets	
Analysis window	256 samples of one annotated heartbeat segment (R-peak located at 100th sample) ⇒ 0.711 s	128 samples of one annotated ADL segment (50% overlap with adjacent segments) ⇒ 2.56 s

**Table 2:** Summary of binary and multiclass HAR performances.

Classification	Binary (X-vs-ALL)						Multiclass	
	**LAY**	**SIT**	**STD**	**WALK**	**UP**	**DOWN**		
Number of features	1	5	11	14	12	15	16	17
Metric’s value	1.0 *	0.822 *	0.804 *	0.867 *	0.861 *	0.804 *	0.877 ^†^	0.872 ^†^
Number of extractors	1	4	5	5	8	7	8	
– with wavelet generators	0	2	4	4	7	7	6	
– with direct integration	1	2	1	1	1	0	2	

* MCC, ^†^ Accuracy.

**Table 3:** Comparison of the multiclass HAR performances.

Model	Accuracy	Input Size	Parameters
CNN [30]	96.98%	1152 (3 × 3 × 128: 3 axes for -acceleration, -ang. velocity, -acceleration w/o gravit. component)	0.342M
CNN [49]	97.21%		0.45M
CNN [50]	96.98%		0.35M
CNN [51]	91.67%		0.424M
CNN-LSTM [51]	94.48%		3.5M
BiLSTM [51]	93.91%		0.168M
iSPLInception [51]	95.09%		1.33M
CNN-BiLSTM [52]	96.37%		0.631M
LSTM-CNN [53]	95.78%		0.049M
CNN+stat. features [54]	97.63%	384 (3 × 1 × 128)	—
4 layer CNN-LSTM [55]	99.39%	768 (3 × 2 × 128)	—
This work: Feedforward NN (1 hidden layer of 20 neurons)	87.72%	16 (features from 3 signals: $acc_{x}$, $acc_{y}$, $gyr_{z}$)	466
	87.17%	17 (features from 3 signals:$acc_{x}$, $acc_{y}$, $acc_{z}$)	486

**Table 4:** Summary of required specifications for hardware implementation.

Component	8 Feature Extractors (6 with Wavelet Generators, 2 with Direct Integration)				ADC
	**Amplifier**	**Analog Mixer**	**Wavelet Generator**	**Integrator**	
Requirements	differential variable gain	4 switches	storage of wavelet configurations programmable clock (50 Hz/360 Hz)	$F_{c}\ll 0.39$ Hz	8-bit precision $F_{c}\leq 180$ Hz

**Table 5:** Performance comparison of amplification stages from state-of-the-art front-end designs.

Reference	[62] (2017)	[65] (2022)	[63] (2022)	[66] (2020)	[67] (2022)	[64] (2023)
Technology (nm)	180	180	65	180	180	180
Area (mm^2^)	0.0228 *^†^	0.07	0.16	0.72 *^†^	0.57 *^†^	0.122 *
Power (µW)	5.04 ^‡^, 5.74 ^§^	81	0.303	2.3 ^¶^, 3 ^∥^	2.17	0.507
Supply voltage (V)	1.4	2.7	0.8	1.8	1	1.2
Gain (dB)	38–72	0–21.6	44–71	42–50	40–53.5	30–45
Input referred noise	2.98 μ$V_{\mathrm{rms}}$ (1–4500 Hz)	150 nV/Hz (at 10 Hz)	1.4 μ$V_{\mathrm{rms}}$ (1–100 Hz)	1.22 μ$V_{\mathrm{rms}}$ (1–100 Hz)	1.16 μ$V_{\mathrm{rms}}$ (0.5–100 Hz)	0.67 μ$V_{\mathrm{rms}}$ (0.5–150 Hz)
Results	Measurement	Simulation	Simulation	Measurement	Simulation	Simulation

* Total chip area, ^†^ Active part, ^‡^ LNA-PGA, ^§^ LNA-PGA-LPF, ^¶^ Instrum. amplifier, ^∥^ PGA-LPF-ADC.

**Table 6:** Performance comparison of first-order LPFs with a sub-Hertz cut-off frequency.

Reference	[68] (2020)	[69] (2018)	[70] (2011)
Technology (nm)	180	180	350
Area (mm^2^)	0.0156	0.051	0.07
Power (µW)	1.08	2.7	0.005
Supply voltage (V)	1.8	1.8	1
*F*_c_ (Hz)	0.22–39.1k	0.114–2.5k	0.002–90

**Table 7:** Best $V_{t2}$, $V_{gc}$ pairs with the lowest $F_{c}$ from AC simulations in the integrator configuration.

V_CM_ (V)	V_t2_ (mV)	V_gc_ (mV)	F_c_ (mHz)	GE_DC_ (dB)
0.9	410	80	191.5	0.495
	470	130	185.5	0.481
	500	150	199.6	0.444
0.6	420	90	184.4	0.490
	470	130	192.8	0.453
	500	150	209.0	0.415

**Table 8:** G_m_-C integrator’s biasing, control voltages, and performance summary.

V_CM_ (V)	V_t1_ (V)	V_c_ (V)	V_t2_ (V)	V_gc_ (V)	V_b1_ (V)	V_b2_ (V)	F_c_ (mHz)	GE_DC_ (dB)	amp_MAX_ (mV)	P (nW)
0.6	0.6	1.1	0.5	0.15	0.6	0.4	211.5	0.410	427	625

## Data Availability

The datasets analyzed in this study are openly available in PhysioNet at https://doi.org/10.13026/C2F305 (accessed on 14 November 2023) and in the UCI Machine Learning Repository at https://doi.org/10.24432/C54S4K (accessed on 14 November 2023).

## Abbreviations

The following abbreviations are used in this manuscript:

IoT	Internet of Things
A2I	analog-to-information
CS	compressive sampling
A2F	analog-to-feature
ML	machine learning
ECG	electrocardiogram
BASN	body area sensor network
DL	deep learning
KNN	K-nearest neighbor
NB	naive Bayes
SVM	support vector machine
DT	decision tree
RF	random forest
CNN	convolutional neural network
LSTM	long short-term memory
NUWS	non-uniform wavelet sampling
HAR	human activity recognition
ADC	analog-to-digital converter
IG	information gain
LDA	linear discriminant analysis
SFS	sequential forward search
NN	neural network
GPU	graphics processing unit
ADL	activity of daily living
MCC	Matthews correlation coefficient
CMOS	complementary metal–oxide–semiconductor
LPF	low-pass filter
ASIC	application-specific integrated circuit
LNA	low-noise amplifier
PGA	programmable gain amplifier
SAR	successive approximation register
OTA	operational transconductance amplifier
PMOS	positive channel metal–oxide–semiconductor
NMOS	negative channel metal–oxide–semiconductor
DC	direct current
AC	alternating current
FoM	figure of merit
FPGA	field programmable gate array
PCA	principal component analysis
